# The association of care satisfaction and COVID-19 contact restrictions with quality of life in long-term care homes residents in Germany: a cross-sectional study

**DOI:** 10.1007/s41999-022-00710-9

**Published:** 2022-10-31

**Authors:** Rieka von der Warth, Boris A. Brühmann, Erik Farin-Glattacker

**Affiliations:** Section of Health Care Research and Rehabilitation Research, Faculty of Medicine and Medical Center, University of Freiburg, Hugstetter Str. 49, 79106 Freiburg, Germany

**Keywords:** Long-term care, Quality of life, Care satisfaction, Covid-19, Contact restrictions, Nursing care

## Abstract

**Background:**

Quality of life (QoL) is a widely recognised outcome in residents of long-term care homes. However, little is known about the impact of care satisfaction on QoL. The aim of this study was to assess the association between care satisfaction and QoL in residents of long-term care homes. Additionally, we were able to assess the impact of the Covid-19 contact restrictions on QoL.

**Methods:**

We applied a cross-sectional study in *N* = 40 long-term care homes in Baden-Wuerttemberg, Germany. Using regression models, we analysed the association between QoL (operationalised through the World Health Organization Quality of Life Assessment—Old Module [WHOQOL-OLD]) and self-rated nursing care satisfaction. The date on which the questionnaire was completed was used to calculate whether the completion was prior the emergence of the Covid-19 contact restrictions. Further potential confounders were included in the analysis.

**Results:**

*N* = 419 residents of long-term care homes participated. Explained variance of QoL was low in our models at 2 to 16%. Self-rated nursing care satisfaction was the strongest predictor of QoL and positively linked to the following subdimensions of QoL: autonomy; past, present and future activities; social participation; intimacy. The Covid-19 contact restrictions were negatively linked to social participation.

**Conclusion:**

Nursing care satisfaction was associated with QoL in residents of long-term care homes. Future research should focus on the direction of the association and different aspects of nursing care satisfaction with QoL. Furthermore, we showed the impact of contact restriction during the Covid-19 lockdown on social participation.

**Trial Registration:**

WHO UTN: U1111–1196-6611; DRKS-ID: DRKS00012703 (Date of Registration in DRKS: 2017/08/23).

**Supplementary Information:**

The online version contains supplementary material available at 10.1007/s41999-022-00710-9.

## Key summary points

**Aim:**

To investigate the association between care satisfaction and quality of life as well as the impact of the Covid-19 contact restrictions on long-term care home residents in Germany.

**Findings:**

While self-rated nursing care satisfaction of long-term care residents was associated with quality of life, medical care satisfaction was not associated with quality of life. Furthermore, we showed that the Covid-19 restrictions had a negative impact on social participation in long-term care residents, which we argue to be due to less social activities.

**Message:**

Quality of life in long-term care residents is an important outcome criterion and this paper gives further implication for clinical practice in long-term care homes.

**Supplementary Information:**

The online version contains supplementary material available at 10.1007/s41999-022-00710-9.

## Introduction

About 3.3 million people required care in Germany in 2017, displaying an increase of 67% in less than 20 years. About 24% of all people in need of care were living in long-term care homes in 2017. At the same time, costs for all kinds of nursing care increased equally from about 5 billion € to 44 billion € between 1999 and 2019 [1]. Thus, the demographic change is in full swing [2], and a further increase in people in need of care and care costs can be expected [3].

Quality of life (QoL) is widely recognised as an important outcome in residents of long-term care homes [e.g. 4]. Different important dimensions of QoL are, among others, physical and psychological domains, the level of independence, environmental factors [5]. QoL is an important outcome in residents of long-term care homes as it is associated with other relevant outcomes in the elderly. For instance, physical domains of health-related QoL were strongly associated with mortality and hospitalizations in older adults [6–10], being an even stronger predictor than multiple biomarkers [6]. On the other hand, hospitalizations were shown to be associated with major health care costs [11, 12]. Thus, knowing possible predictors for QoL is of clinical relevance.

Known predictors for reduced QoL in long-term care home residents are, among others, pain, sleep difficulties and younger age [13]. Furthermore, severity of cognitive impairment and physical functioning were found to be associated with a decrease in QoL in people with cognitive impairment [14, 15]. High engagement in social activities was also described to have a positive influence on QoL in people with dementia living in long-term care homes [16, 17].

Little is known about the impact of the Covid-19 pandemic on long-term care residents in Germany yet. However, as previous studies have described the positive impact of visiting relatives on QoL [14, 15] and the global Covid-19 pandemic has led to contact restrictions in long term care homes in many countries [1, 18], a decrease in QoL can be hypothesised.

Additionally, quality of care itself was associated with QoL in patients with dementia [19]. Care-related factors that are associated with QoL are, for example, the availability of geriatricians in a long term care home [15]. The evidence on the correlation between the quality of care and QoL remains divided. While some research found a correlation between QoL and quality ratings [20], another study did not find such correlations [21]. Furthermore, care satisfaction should also be considered as predictor of QoL, as previous studies observed small correlations of QoL and care satisfaction, arguing the two concepts to be different [22].

However, as evidence on the link between care satisfaction, quality of care, and QoL is small [23], this study aims to provide a further insight into the association of self-rated care satisfaction and self-rated QoL in long-term home residents in Germany. Furthermore, we assessed the impact of the Covid-19 restrictions on QoL as a confounding variable with high relevance to QoL long term care home residents.

## Material and methods

This cross-sectional study was conducted at the University of Freiburg—Medical Faculty. Ethics Approval for this study was granted by the ethics committee at the Chamber of Physicians of the State of Baden-Württemberg (Reference No. B-F-2017-127) as well as by the ethics committee at the University of Freiburg Medical Centre (Reference No. 333/17). The study was registered at the German Clinical Trial Register (DRKS00012703) and funded by the Innovation Committee at the Federal Joint Committee (G-BA), Germany (Grant No. NVF1_2016-080). The reporting of this study is in line with the STROBE checklist [24].

### Participants and procedures

Residents of long-term care homes were asked to answer a paper–pencil questionnaire between August 2018 and August 2020. In total, residents from *N* = 40 long-term care homes in the federal state of Baden-Wuerttemberg, Germany, took part in this study. Recruitment was part of a greater study, assessing the impact of a complex health services intervention in long-term care homes [25]. As part of this, a local nurse informed potential participants about the study. Furthermore, written study information was handed out, describing the study design, the voluntary nature of the study, and data protection regulations. A written consent form was provided in duplicate, one to hand in for the study, and one to keep for the resident.

All residents were asked to participate unless they met the exclusion, defined as a diagnosis for dementia and being a resident of the long-term care home for less than three months. The nursing staff were asked to respond to the residents' questions regarding the questionnaire and items, and to motivate them into answering the questionnaire but were not supposed to assist them in answering the questionnaire itself. Any relatives on-site were allowed to help the resident with answering, but again were asked not to influence a resident’s assessment. Whether a relative or nurse provided assistance was documented on the questionnaire.

### Measures

QoL was measured using the German version of “The World Health Organization Quality of Life Assessment-Old Module” (WHOQOL-OLD) [26, 27]. The assessment comprises five scales: Sensory Abilities; Autonomy; Past, Present and Future Activities; Social Participation; Death and Dying; Intimacy. Internal consistency was found to be between *α* = 0.63 and *α* = 0.89 using Cronbach’s Alpha [27]. Mean scores range from 1 to 5, with a higher score indicating a better QoL.

To assess nursing care satisfaction, an adapted version of the “Relatives Screening Questionnaire Assessing Satisfaction” (ZUF-A-7) [28] was used. The screening was originally designed for relatives to rate their satisfaction with the nursing care. We adapted the wording of the items so that residents could answer them themselves (*e.g. Do you feel that the nursing staff cares enough for you?; Are you getting the care you want?*). The screening consists of seven items and the score ranges from 7 to 28, with a higher score indicating greater satisfaction. Cronbach’s Alpha in this study was *α* = 0.86.

Medical care satisfaction was assessed using a single item: “How satisfied are you with medical care in the long-term care home overall?”. The score ranges from 1 to 4, with one indicating “very dissatisfied” and four indicating “very satisfied”.

Assessment of sociodemographic data included gender, an ordinal assessment of the age (8 categories), visits of relatives (*yes; no; I don’t have any relatives*), and an ordinal assessment of the time the participants have already been living in the long-term care home.

Additionally, the day on which the questionnaire was completed was recorded. This information was used to calculate whether the questionnaire was completed prior to the emergence of the Covid-19 pandemic. We used 15th March 2020 as a reference day, as the first official national lockdown in Germany was announced in the middle of March, 2020. As a result, visits by relatives were largely prohibited [1].

Before the start of the study, a methodological test was carried out. For this purpose, the questionnaire was presented to *N* = 9 residents in a one-on-one cognitive pre-test. Two methods were combined: "thinking aloud" and "asking questions" [29]. The aim of the cognitive interviews was to test all the intended items for comprehensibility and acceptability for elderly residents of long-term care homes.

### Statistical analyses

The association of care satisfaction and QoL was analysed by conducting multiple hierarchical regression analyses. Predictors were included in three blocks in the analyses, to assess incremental explanation of variance using the forced entry option. The blocks included sociodemographic data (age, gender), living situation (visiting relatives, time living at the long-term care home, the first Covid-19 lockdown (15th March 2020)) and satisfaction (nursing care satisfaction, medical care satisfaction). As we had included several nominal and ordinal scales as predictors, which might cause multicollinearity, we decided to conduct a preliminary analysis to select the most important predictor categories per outcome. For this purpose, bivariate correlations were conducted between each outcome and each predictor. Only predictors with a *p* value < 0.200 were included in the main analysis (see Supporting Information 1 for the results of the bivariate correlations). Nominal and ordinal variables were dummy coded before all analyses. Assumptions for the regression models (linearity, collinearity, homoscedasticity, normality of residuals) were tested by checking the VIF-value, the Durbin–Watson test, as well as plots of *ZRESID against *ZPRED and normal *P*–*P* plots. Missing values were excluded listwise. Analyses were conducted using IBM’s SPSS Statistics Version 27 [30].

## Results

### Sample

Of *N* = 2155 questionnaires sent out to *n* = 53 nursing homes, a total of *N* = 419 residents from *n* = 40 nursing homes completed the questionnaire. Reasons for non-participation are unknown, but could be both, no interest or not meeting inclusion criteria. *N* = 285 participants were female (68%), and 71% of all participants were 80 years or older. About half of the participants stated to have been living longer than two years in their respective long-term care home. A majority stated that they had received help from someone while filling out the questionnaire (81%). See Table 1 for a full overview.

**Table 1:** Sociodemographic data of participants

	*N*	%
*Gender*		
Male	132	31.7
Female	285	68.3
*Nationality*		
German	406	98.5
Other	6	1.5
*Age group*		
Up to 59 years	21	5.0
60 to 64 years	16	3.8
65 to 69 years	21	5.0
70 to 74 years	16	3.8
75 to 79 years	46	11.1
80 to 84 years	88	21.2
85 to 89 years	97	23.3
90 years and older	111	26.7
*Visits of relatives*		
Yes	366	87.8
No	38	9.1
I don’t have any relatives	13	3.1
*Time living in the long-term home*		
Less than 6 months	38	9.4
6 months to 1 year	55	13.6
1 to 2 years	108	26.7
More than 2 years	203	50.2
*Covid-19 Lockdown (March 15 2020)*		
Before the first lockdown	321	79.3
After the first lockdown	84	20.7
*Help from relatives/nurse completing the questionnaire*		
Yes	317	81.3
No	73	18.7

Mean score for nursing care satisfaction at the long-term care home was 24.6 (SD = 3.4; Min–Max: 7–28), while mean medical care satisfaction was 3.2 (SD = 0.8; Min–Max: 1–4).

### Quality of life

The average QoL on each of the scales was close to 3.5, with the subscale Autonomy showing the lowest mean score of 3.38 (SD = 0.85). Participants reported the highest QoL on the subscale of Death and Dying, with an average of 3.73. However, the standard deviation was also the highest on this scale (SD = 1.05), describing that there was the biggest difference in perception among participants. See Table 2 for all mean scores of QoL.

**Table 2:** Mean Quality of life assessed using “The World Health Organization Quality of Life Assessment—Old Module” [26]

	Mean	SD	Min–Max
Sensory abilities	3.45	0.99	1–5
Autonomy	3.38	0.85	1–5
Past, present and future activitiy	3.59	0.73	1.25–5
Social participation	3.44	0.87	1–5
Death and dying	3.73	1.05	1–5
Intimacy	3.50	0.92	1–5

### Associations with quality of life

Explanation in variance of QoL was low to moderate in our analyses, with a 2% explanation of variance in Death and Dying and 16% in Intimacy. We found no significant association between the QoL domain of Death and Dying and included independent predictors.

Female gender was associated with higher perceived Intimacy (*β* = 0.157; *p* = 0.001). Three age categories were linked to Sensory Abilities. We found that participants within the age group of between 60 and 64 years and between 75–79 years reported better Sensory Abilities (*β* = 0.108; *p* = 0.035 and *β* = 0.105; *p* = 0.043, respectively). Those aged 90 years or older were associated with worse Sensory Abilities (*β* = − 0.117; *p* = 0.026).

Participants, who stated that they had no relatives who would visit them, reported a better Social Participation (*β* = 0.119; *p* = 0.022). Furthermore, participants who had lived at the long-term care home for at least six months but less than a year stated having a lower feeling of Autonomy compared to the other groups (*β* = − 0.103; *p* = 0.045). The first Covid-19 lockdown in March 2020 was negatively linked to Social Participation (*β* = − 0.129; *p* = 0.013).

Adding nursing care satisfaction and medical care satisfaction, the incremental explanation of variance differed between 1% in Death and Dying and 10% in Past, Present and Future Activities. Medical care satisfaction showed no significant association with QoL in any model. Nursing care satisfaction was positively associated with: Autonomy (*β* = 0.267; *p* < 0.001); Past, Present and Future Activities (*β* = 0.330; *p* < 0.001); Social Participation (*β* = 0.285; *p* < 0.001); Intimacy (*β* = 0.311; *p* < 0.001). See Table 3 for a full overview of the regression models.

**Table 3:** Regression models describing the association between included variables and quality of life

	Sensory abilities	Autonomy	Past, present and future activities	Social participation	Death and dying	Intimacy
	*β*	*β*	*β*	*β*	*β*	*β*
Gender		.101				.157**
*Age group*						
Up to 59 years						
60 to 64 years	.108*					− .048
65 to 69 years	.088	.101	.080	.054		
70 to 74 years						
75 to 79 years	.105*					
80 to 84 years			− .029		− .080	
85 to 89 years		− .058				
90 years and older	− .117*		.080		.060	.043
*Visits of relatives*						
Yes	− .053					.045
No	.005					− .113
I don´t have any relatives				.119*		
*Time living in the long-term home*						
Less than 6 months				− .073		
6 months to 1 year		− .103*				
1 to 2 years						
More than 2 years				.043		
Covid-19 Lockdown (March 15 2020)				− .129*		
Satisfaction with nursing care		.267***	.330***	.285***	.063	.311***
Satisfaction with medical care		.031	.014	− .005	.072	− .039
*R*^2^/*R*^2^ corrected	.06/.05	.12/.10	.14/.12	.13/.12	.03/.02	.17/.16

Empty cells occure when predictor is not part of the regression model as the significance level was lower that *p* < .200 in the preliminary analyses; *β* = *standardised Beta*

*p < 0.05

**p < .0.01

***p < 0.001

## Discussion

We conducted a cross-sectional study, assessing the association of care satisfaction with QoL in long term care home residents in Germany. Furthermore, we were able to assess the impact of the contact restriction as part of the Covid-19 pandemic on QoL.

Mean QoL was slightly lower in most domains in our study than in previous studies on the QoL of the elderly [26]. Yet, participants in our study reported higher QoL in the scale of Death or Dying compared to previous research [26, 31], with participants in this study being less concerned about their own death. Another study assessing QoL in people living in long-term care homes also found the highest values for Death and Dying [32]. Yet, the WHOQOL-OLD is validated for community-dwelling older individuals and thus, the differences to the validation study by Power, Quinn [26] are not surprising. Furthermore, QoL seems to be generally lower in people living in long-term care homes compared to elderly people receiving home care [14, 33].

While Sandgren, Arnoldsson [32] found no differences in QoL between gender and age groups, female gender was associated with higher perceived Intimacy, and younger age seemed to be associated with better Sensory Abilities in this study. Another study equally reported higher scores in Intimacy for female participants but better Sensory Abilities with increasing age [34], even though this study was not conducted in long-term care homes. Also, younger age was comparably shown to predict QoL in long-term care homes [13].

Surprisingly, we found that those participants who stated having no relatives reported a higher QoL in Social Participation. Visiting relatives were previously reported to predict high QoL [15]. However, results on the impact of relatives on QoL needs more differentiated discussion. For instance, whilst being married and having a spouse helping seems to have a positive impact, a negative link between having children and QoL was found [13, 34, 35]. Yet, only 13 individuals stated to have no relatives in our study and the interpretability of the results is reduced.

Additionally, the appearance of Covid-19 had a negative impact on Social Participation in our study. Previous research showed that 64% of all relatives expected a decrease in QoL of long-term care residents [36]. Similar results were reported during the SARS outbreak in 2003, when relatives expressed their concerns on the well-being of long-term care residents [37]. A systematic review concluded a reduced QoL as a result of e.g. increase in anxiety, depression and cognitive symptoms in patients with dementia [38]. We would argue, in line with Lapid, Koopmans [18], that the Covid-19 pandemic had an impact on QoL due to contact restrictions in long-term care homes. However, contact restrictions were not limited to relatives visiting, but also had an impact on resident’s social engagement, such as having contact with each other and activities within the long-term home set up by nursing staff and volunteers. Social engagement was not only to be shown to predict QoL in residents of long-term homes [39], but residents having a high engagement showed lower mortality [40]. In addition, having the possibility to go outside, was shown to be an important predictor of QoL [39]. Recently, the architectural and design malfunctioning in long-term care homes revealed by the Covid-19 pandemic, resulting in a negative impact on residents QoL is discussed in the literature [41]. In the following articles describing what pandemic learnings can be used to improve resident centred nursing emerged [19, 42, 43]. For instance, QoL could be improved by using technical devices to communicate in e.g. residential forums or conversation cafes, but also activities such as religious and cultural practices or exercises could be maintained by using telephone or videoconference [43]. However, future research should examine the complex relationship between visiting relatives, social engagement and QoL in long term care residents.

Furthermore, and also surprisingly, living less than one year at the long-term care home was linked to less autonomy. Autonomy was found to be a major need for older people receiving home care [44]. Thus, we would hypothesise that residents only living in the long-term care home for a shorter period perceive a decrease in Autonomy, which normalises over time. However, a longitudinal assessment of QoL and Autonomy would be needed to answer this hypotheses.

Nursing care satisfaction was the strongest predictor of QoL in all models, except for Sensory Abilities. Comparably to previous research, the association between care satisfaction and QoL were statistically weak [22] with a small incremental explanation of variance. However, compared to the study by Lalic, Wimmer [22], we controlled for gender, age and other variables. As our assessment of nursing care satisfaction was generic, future research should focus on single aspects of care satisfaction. For instance other authors identified themes like improving health and healthcare, supporting a good end of life, promoting a positive culture and other aspects of good quality in care [23, 45] that should be considered.

### Strength and limitation

Our study gave further insight into the association of self-rated satisfaction with care and self-rated QoL in long-term care home residents in Germany. Furthermore, the impact of the Covid-19 restrictions on QoL was investigated. We were able to reach a comparably high sample size with *N* = 419, in a multicentre study assessing the QoL of residents in multiple long-term care homes.

One important limitation is the cross-sectional approach of our study. Firstly, the approach gives no insight into the direction of the association and it remains unclear whether a high care satisfaction has an impact on QoL or if the association is bi-directional. Furthermore, due to the cross-sectional approach we cannot exclude the appearance of a common method variance and thus an overestimation of the association between QoL and care satisfaction.

As dementia was an exclusion reason, we did not further control for cognitive impairments in our study. Additionally, it is unclear how many residents did not take part in our study due to possible cognitive impairment, as we have no information on non-response rates. Thus, a sample bias must be assumed. Additionally, between 34 to 50% of all long-term care home residents show a diagnosis of dementia in Germany [46]. Thus, a high proportion of residents were excluded from this study, which might further affect the results.

Furthermore, we did not combine the WHOQOL-OLD with the WHOQOL-BREF [47], as recommended [26]. We did this, as we did not want the questionnaire to be too long and exhausting for residents in long-term care homes. The cognitive pre-test, which we laid out before the start of the study, supported this approach. Lastly, as the cognitive pre-test showed that it was easier for participants to answer questions with a choice of answers instead of free text entries, we assessed naturally metric variables (age, time living in the long-term care home) as ordinal scaled items. Also the distribution between groups was unequal, which might lead to an underestimation, e.g. in the impact of the Covid-19 contact restrictions on QoL. One further concern might be, that relatives or nurses were allowed to assist the answering of questionnaire and thus, social desirability might be problem. However, we conducted an exploratory analysis, which showed no differences in QoL between those residents who received assistance and those who did not. Yet, reproducibility of our findings should be examined and future research should test the impact of relatives and nurses assisting.

Additionally, we did not find any predictive value of medical care satisfaction. This might be because we assessed medical care satisfaction by a single item and thus, the predictive value of it might be small due to methodological issues.

## Conclusion

With our study, we were able to give further insight into the relationship between care satisfaction and QoL in residents of long-term care homes. Results of this paper indicate further implication for clinical practice in long-term care homes. First of all, quality of life might be increased by providing social activities. This could be reached by a sufficient nursing staff to long-term care resident ratio as well as good working conditions. By this, nursing staff would have the capacity to organise social activities and in general spend time with residents. Furthermore, self-rated care satisfaction is important and should be considered as quality criterion.

In detail, we found nursing care satisfaction to be the strongest predictor for QoL. However, future research should focus on different aspects of care satisfaction and the association with QoL. Furthermore, we were able to show the impact of contact restrictions during the Covid-19 pandemic on social participation. As having relatives showed no correlation, we argue that these impairments are due to less social activity and contacts within the long-term care home.

## Supplementary Information

Below is the link to the electronic supplementary material.

Supplementary file1 (PDF 338 KB)

## Data Availability

Due to the nature of this research, participants of this study did not agree for their data to be shared publicly. Thus, data is not available publicly.
