# Supplementary material for: The association of care satisfaction and COVID-19 contact restrictions with quality of life in long-term care homes residents in Germany: a cross-sectional study
Source: Eur Geriatr Med. 2022 Oct 31;13(6):1335–42. doi: 10.1007/s41999-022-00710-9 (PMC9628361; doi:10.1007/s41999-022-00710-9)
Supplement: Supplementary file 1 — Supplementary file1 (PDF 338 KB) [file 41999_2022_710_MOESM1_ESM.pdf]

Supporting Information 1: Bivariate correlations to select predictors for regression models

= Included in regression model

Bivariate correlations between predictors and outcomes								
		Sensory Abilities	Autonomy	Past. Present and Future Activities	Social Participation	Death and Dying	Intimacy
Satisfaction with nursing care	Pearson-Correlation	.032	.306**	.354**	.263**	.087	.330**
	p-Value	.535	<.001	<.001	<.001	0.094	<.001
	N	385	357	368	364	373	384
Gender	Pearson-Correlation	.004	.087	.040	.059	-.029	.201**
	p-Value	.932	.100	.444	.257	.574	<.001
	N	389	359	372	368	377	388
Age –up to 59 years	Pearson-Correlation	.000	.066	.004	.026	-.051	-.049
	p-Value	.993	.210	.940	.616	.319	.340
	N	389	359	372	368	377	388
Age – 60 to 64 years	Pearson-Correlation	.119*	.021	-.043	-.056	.029	-.124*
	p-Value	.019	.687	.404	.283	.571	.015
	N	389	359	372	368	377	388
Age - 65 to 69 years	Pearson-Correlation	.106*	.136**	.106*	.080	.026	.000
	p-Value	.037	.010	.042	.125	.613	.995
	N	389	359	372	368	377	388
Age - 70 to 74 years	Pearson-Correlation	.055	.010	-.043	.017	.040	-.060
	p-Value	.282	.853	.404	.747	.437	.237
	N	389	359	372	368	377	388
Age - 75 to 79 years	Pearson-Correlation	.123*	-.025	-.066	-.062	-.033	-.026
	p-Value	.016	.639	.201	.235	.522	.611
	N	389	359	372	368	377	388
Ages - 80 to 84 years	Pearson-Correlation	.021	-.041	-.074	-.014	-.094	.027
	p-Value	.683	.434	.156	.784	.069	.598
	N	389	359	372	368	377	388
Age - 85 to 89 years	Pearson-Correlation	-.062	-.080	-.008	-.031	.001	-.031
	p-Value	.226	.132	.880	.556	.978	.544
	N	389	359	372	368	377	388
Age - 90 years and older	Pearson-Correlation	-.176**	.024	.108*	.053	.089	.126*
	p-Value	<.001	.655	.036	.315	.086	.013
	N	389	359	372	368	377	388
	Pearson-Correlation	-.012	-.012	-.058	-.084	-.003	.021

Time living in the long-term home – less than 6 months	p-Value	.823	.821	.272	.114	.948	.688
	N	379	352	364	359	365	377
Time living in the long-term home – 6 month to 1 years	Pearson-Correlation	.000	-.088	.033	-.051	.055	.008
	p-Value	.996	.100	.528	.333	.292	.884
	N	379	352	364	359	365	377
Time living in the long-term home – 1 to 2 years	Pearson-Correlation	.057	-.002	.025	.009	-.010	-.057
	p-Value	.266	.970	.631	.864	.856	.271
	N	379	352	364	359	365	377
Time living in the long-term home – More than 3 years	Pearson-Correlation	-.044	.068	-.011	.078	-.027	.033
	p-Value	.398	.205	.834	.140	.604	.521
	N	379	352	364	359	365	377
Visits of relatives - yes	Pearson-Correlation	-.126*	.067	-.005	-.032	.033	.194**
	p-Value	.013	.206	.927	.536	.528	<.001
	N	389	359	372	368	377	388
Visits of relatives - No	Pearson-Correlation	.118*	-.065	-.034	-.042	-.039	-.198**
	p-Value	.020	.216	.515	.419	.449	<.001
	N	389	359	372	368	377	388
Visits of relatives – I don´t have any relatives	Pearson-Correlation	.044	-.018	.066	.131*	.003	-.036
	p-Value	.392	.737	.201	.012	.954	.475
	N	389	359	372	368	377	388
Covid-19 Lockdown (March 15 2020)	Pearson-Correlation	.044	-.059	-.003	-.128*	.014	.002
	p-Value	.389	.274	.960	.015	.784	.964
	N	378	348	363	359	367	377
Satisfaction with medical care	Pearson-Correlation	0.062	.132*	.128*	.072	.096	.084
	p-Value	0.229	.013	.014	.172	.064	.100
	N	384	358	368	362	373	384

*. The correlation is significant at the 0.05 level (2-sided).
**.The correlation is significant at the 0.01 level (2-sided).
